# Towards a fine-scale picture of European genetic diversity

**DOI:** 10.1038/s41431-020-0620-1

**Published:** 2020-04-01

**Authors:** Michael Nothnagel

**Affiliations:** 1 Cologne Center for Genomics, University of Cologne, Cologne, Germany; 2 University Hospital Cologne, Cologne, Germany

**Keywords:** Population genetics, Genetics

Paintings on the cave walls of Chauvet, Lascaux, and other sites bear witness to the presence of modern humans within France’s borders over the past 35,000 years, rendering this country an exciting place to do human population genetic studies. However, France’s finer genetic structure has so far been described only with respect to selected geographic regions. Saint Pierre et al. [1] fill this gap with the first comprehensive large-scale study that looks at all French regions. Their analysis is based on autosomal single-nucleotide polymorphism chip data from two independent large cohorts comprising nearly 2200 individuals in total. The authors report on the existence of five to six population clusters that closely correspond to geographic, historic, and linguistic divisions of the country, which partially follow larger rivers and mountain ranges. Also, a broad Northeast-Southwestern divide becomes apparent. Interestingly, Saint Pierre et al. observe varying contributions to these clusters by past hunter-gatherers, Neolithic farmers, and Steppe pastoralists, respectively, under a simple three-sources model. Finally, the authors trace population sizes in the past 4500 years, observing the onset of exponential growth in the late Bronze Age around 3000 years (100 generations) ago and a severe dip in size during the time when the Black Death haunted Europe.

After a first wave of papers describing the broad genetic structure of European populations with respect to common variants in 2008, namely the papers by Lao and Lu et al. [2], Novembre et al. [3], and Heath et al. [4], this study by Saint Pierre et al. significantly adds to a second most recent set of population genetics studies based on next-generation sequencing technologies. In the past few years, numerous publications have reported on the fine-scale genetic structure of particular countries, including, for example, the United Kingdom and Ireland, the Netherlands, the Iberian Peninsula, Sweden, Italy, and now France. While the Western part of the continent as well as selected regions in the South and North have, thus, become well characterized, the lack of fine-scale studies on Central European countries, notably including the German speaking and Slavic ones, but also those in Southeastern Europe, is striking and leaves the pan-European picture incomplete. Limited resources may be an explanation for this observation in some countries, possibly including Eastern European ones, but definitely not for Germany, Austria, and Switzerland. With respect to basic medical research and health care, but also archeological research, future commercial applications and innovative industries, these countries would be well advised to invest heavily in their human genetic research in the years to come and to complete the picture of European genetic diversity.

The subtle (and not so subtle) differences demonstrated by this study in the genetic makeup of France’s population are of importance for future studies. While population stratification may be well controlled in common-variant population-based association studies, this is not necessarily the case when studying rare variants. In fact, the broad genetic divide between Northeastern and Southwestern France, coupled with different environmental exposures and perhaps different lifestyle habits, may turn out to be a prime example for this concern in future rare-variant studies. As theoretically investigated by Mathieson and McVean [5] nearly a decade ago, standard methods may prove to be unable to adequately adjust for the effects of population stratification when systematic nongenetic confounding factors are present yet are restricted to small geographic regions. Those future studies will have to pay attention to this confounding potential, perhaps by restricting sampling to geographic regions housing only one of the clusters identified in the study by Saint Pierre et al.

This study also exposes the often-strong dependency of the analysis results from genetic studies on the particular sample set at hand, in particular for those that focus on rare variation. While both study cohorts, comprising close to 800 and about 1400 samples, respectively, yielded broadly concordant results, there were numerous differences in the details. The application of identical statistical approaches to two different cohorts covering the same geographic area is one of the particular strengths of this study in that, it well illustrates the impact of sampling variance, while also providing an assessment of the robustness of the obtained results. This may serve as a warning against overinterpretation of results when only one data set is available, which is naturally the case for most population genetic studies. Notably, this also applies to studies based on ancient DNA which are very often characterized by an extremely sparse sampling in both time and space and by limited genetic data.

Estimating proportions of genetic heritage from primordial European hunter-gatherers, distant Steppe people and migrating early farmers and assessing population size fluctuations are fascinating endeavors and offer glimpses into our past. However, such events may also resemble a distant mirror of our future. Given the comparatively large genetic homogeneity of humankind, today’s interconnected world is more at risk than ever to see Black Death-style pandemics occur, this time on a global rather than a local scale and at higher frequency. Furthermore, this study reminds us that events dating back to medieval times, the Bronze and even the Stone Age significantly contributed to shaping the genetic structure of the French and other extant populations. Such events are therefore still relevant to human genetic research. Finally, the study results yield further evidence that all extant human populations are the product of multiple admixture events during their formation. A population that would have developed independently from all others from the start of humankind would be a very rare exception, if such a population exists at all.
